# Social enterprise interventions to tackle food insecurity in Sub-Saharan Africa: a scoping literature review

**DOI:** 10.3389/fpubh.2025.1604405

**Published:** 2025-08-18

**Authors:** Victoria von Salmuth, Thomas Phillips, Anindita Bhattacharjee, Albert Kwansa, Robert Akparibo

**Affiliations:** ^1^Department of Family Medicine, CAPHRI, Maastricht University, Maastricht, Netherlands; ^2^Faculty of Health, Medicine and Life Sciences, Maastricht University, Maastricht, Netherlands; ^3^Division of Population Health, School of Medicine and Population Health, University of Sheffield, Sheffield, United Kingdom

**Keywords:** social enterprise, community support groups, food security, Sub-Saharan Africa, review

## Abstract

**Background:**

Food insecurity remains a significant global challenge, particularly in low- and middle-income countries (LMICs), where it contributes to the growing prevalence of the double burden of malnutrition. Social enterprises (SEs) are increasingly recognized as effective tools for addressing social challenges through innovative and sustainable approaches. However, their role in mitigating food insecurity in Sub-Saharan Africa has not been extensively explored. This scoping review seeks to assess the scope, depth, and impact of existing research on SE interventions aimed at addressing food insecurity in Sub-Saharan Africa.

**Methods:**

We conducted searches across six academic databases, Medline, EMBASE, CINAHL, Scopus, Web of Science, and Google Scholar, to identify peer-reviewed studies evaluating social enterprise (SE) interventions addressing food insecurity in Sub-Saharan Africa. Additional relevant studies were identified by reviewing citations and references from the initial search results. The selected literature was screened, and data were extracted by two independent reviewers. To ensure accuracy, a third reviewer verified the data extraction process.

**Results:**

Overall, 10 articles were included in this review. The identified SE interventions were categorized into three groups: (1) livestock production and supply, (2) microcredit and loan ventures, and (3) biodiversity and education programs.

**Conclusion:**

Social enterprises encompass a promising way forward in contributing to tackle food insecurity in SSA. SEs often work in cooperation with other organizations such as community support groups, Non-Governmental Organizations (NGOs), and governmental organizations. The sustainability of such interventions depends on financial viability, local ownership, adaptability, integration with local systems, and effective monitoring and evaluation.

## Introduction

Food insecurity and malnutrition remain a major challenge globally, especially in low- and middle- income countries (LMICs). By the end of 2023, an estimated 733 million people worldwide, translating into 1 in 11 people suffered from hunger (1). Taking the mid-ranges, this is an increase of 152 million people globally compared to 2019. Africa faces the largest estimated population facing hunger – 20.4 percent, almost 300 million people (2). Correspondingly, in 2023 an estimated 2.33 billion people were moderately or severely food insecure or without access to adequate food (1, 2).

Food security refers to the dietary needs for a productive and healthy life. To achieve food security the FAO defines a situation that exists when all people, at all times, have physical, social and economic access to sufficient, safe and nutritious food that meets their dietary needs and food preferences for an active and healthy life (2). Recent events, including the COVID-19 pandemic and the conflicts between Ukraine and Russia as well as Palestine and Israel from 2020 to 2024, have disrupted national and international food supply chains.

There is a growing consensus in the literature on the need for comprehensive food and nutrition interventions to address the escalating burden of food insecurity (3). However, implementing and funding these interventions remains a significant challenge, particularly in low- and middle-income countries (LMICs) with limited resources (4, 5). Given the multifaceted nature of food insecurity, which is deeply intertwined with various socio-economic factors, it is recommended that interventions go beyond the nutrition sector to address broader social issues (3, 6). While the role of social enterprises in mitigating food insecurity is recognized in many LMICs, the literature provides limited clarity on the potential impact of these interventions.

A preliminary review of existing studies highlights a gap in comprehensive reviews assessing the scope and magnitude of research on the role of social enterprise interventions in addressing food insecurity. Although some evaluation reports are available, no review has systematically examined the extent and nature of evidence on this topic. This review is therefore crucial as it aims to enhance our understanding of the nature, scope and volume of studies evaluating the impact of Social Enterprise (SE) interventions to tackle food insecurity in SSA. The findings will be instrumental in determining whether a more rigorous systematic review is needed to further analyze the strength and quality of the evidence. In doing so, this review will contribute to the existing body of knowledge by mapping the current evidence base, identifying key gaps, and informing future research, policy, and practice related to the role of SE in addressing food insecurity in Sub-Saharan Africa.

In this review, we define social enterprises as organizations with a dual mission of achieving both financial sustainability and a social purpose (3). These enterprises utilize hybrid business models, operating independently or in collaboration with governmental and international agencies, where the income generated is reinvested to drive fundamental changes within social systems (4, 5).

## Materials and methods

### Design and setting

We employed a scoping literature review methodology to explore the existing body of research on SE interventions addressing food insecurity in SSA. It also aimed to identify and analyze the factors influencing the implementation of these interventions.

### Review framework

The review followed the framework described by Arksey and O’Malley (6). This framework outlines 5 key steps, described below, for conducting scoping literature reviews.

#### Step 1: defining the review questions

Following preliminary reading and scoping searches in Medline and Google Scholar, we identified a notable gap in evidence on both the implementation of these interventions and the factors that could influence their success in the global South, particularly in SSA countries. To address this gap, we formulated the following research questions to guide our investigation into the volume, nature, extent, and impact of the literature on this topic:

1. What social enterprise interventions have been implemented in SSA to mitigate food insecurity within the general population.
2. What contextual factors are likely to influence the successful implementation of such social enterprise interventions to achieve impact?

#### Step 2: identifying relevant studies

We developed a search strategy and conducted a pilot scoping search in Medline to test its effectiveness in locating relevant literature. Based on the results, we refined the search strategy to enhance its sensitivity. The finalized search strategy was then employed to perform a comprehensive literature search in six academic databases: Medline, EMBASE, CINAHL, Scopus, Web of Science, and Google Scholar.

To ensure a thorough review, we also conducted supplementary searches, including citation tracking and reviewing reference lists from included studies, to identify additional relevant studies that may have been missed in the initial database searches.

Recognizing the importance of gray literature in addressing complex research questions, we consulted key gray literature sources, including the websites of the Food and Agriculture Organization (FAO), the World Food Programme (WFP), and various national and international NGOs/INGOs focusing on food security issues in SSA. The searches were conducted in June 2023 and updated in July 2024.

Table 1 summarizes the search strategy used to identify peer review published literature in the academic databases. The search was guided by the PICOS framework—Population, Interventions, Control, Outcomes of Interest, and Setting/Context. Social enterprises were defined to include community-oriented organizations, community benefit organizations, charity organizations or non-governmental organizations/NGOs or non-for-profit organizations, community foundation or interest group, and community support group. Interventions designed and delivered by these enterprises, either independently or in collaboration with entities such as government agencies, were considered eligible for inclusion.

**Table 1:** Literature search strategy.

Population	Women OR pregnant women OR mothers, breastfeeding women OR men OR children and adolescents.
Intervention	Any intervention to tackle food insecurity delivered solely, or in partnership, by social enterprises OR community-oriented organizations OR community benefit organizations OR Charity Organizations OR NGOs OR non-governmental organizations OR Non-for-profit organizations OR community foundation OR community support group OR community Interest group.
Controlled	Any other intervention comparing with the above or no intervention
Outcomes	Identification and classification of social enterprise interventions. Number and types of social enterprise interventions that addressed food insecurity. Identification and description of barriers to intervention delivery, e.g., financial, social-political, policies, other contextual barriers. Prevalence of food insecurity: measured using any of the validated food insecurity assessment tools to assess of the four food insecurity dimensions: food access, food availability, food utilization. Secondary outcomes: improvement of livelihoods of households (income family or per capita, etc.…).
Setting	SSA OR Sub-Saharan Africa

#### Step 3: study selection

The selection of studies based on our inclusion criteria (Table 2) was conducted in three stages: title screening, abstract screening, and full-text review, following removal of duplicate studies and reports. Two independent reviewers (VVS and TP) applied a predefined checklist to assess the relevance of the titles and abstracts. In the case of disagreements, resolutions were achieved through discussion, or a third reviewer (RA or AL) was consulted to independently verify the selection. Full texts articles and reports were subsequently downloaded and reviewed by all authors to determine the final set of studies included in the review. If a reviewer was uncertain about the inclusion of a full text during data extraction, it was re-evaluated by a second reviewer (RA) and discussed in a consensus meeting. Table 2 outlines the inclusion criteria employed for the selection of studies.

**Table 2:** Literature selection inclusion and exclusion criteria.

Inclusion criteria	Exclusion criteria
Studies were included if they focus on:	Studies were excluded if they focus on:
Social enterprise interventions	Non-social enterprise interventions
Examined barriers and enablers of social enterprise intervention delivery	Assessed only individuals’ perceptions and experiences of delivering interventions
Aimed to address food security in the general population, including men, women, and children	Not focused on human population
Conducted in Sub-Saharan Africa	Interventions delivered outside of SSA
Conducted and published in English and/or Spanish.	Non-English or Spanish papers
Conducted between 2000 and July 2024	Published before 2000
Paper available online in full text	Non-available in full texts

#### Step 4: charting the data

A standardized data extraction form was developed and agreed upon for the purpose of systematically extracting relevant data from the included studies. Two reviewers (VV and AK) independently applied this form to extract data. Any discrepancies between the extracted data were resolved through discussion during a meeting involving all members of the review team. The key information extracted included the study’s author, year of publication, country of origin, study design, population characteristics (including gender and age), study methods (sampling and data collection methods, primary results or findings, and the authors’ conclusion).

#### Step 5: collating, summarizing, and reporting results

A synthesis and thematic analysis were conducted on the extracted data to provide a structured summary of the findings.

## Results

**Figure 1:**
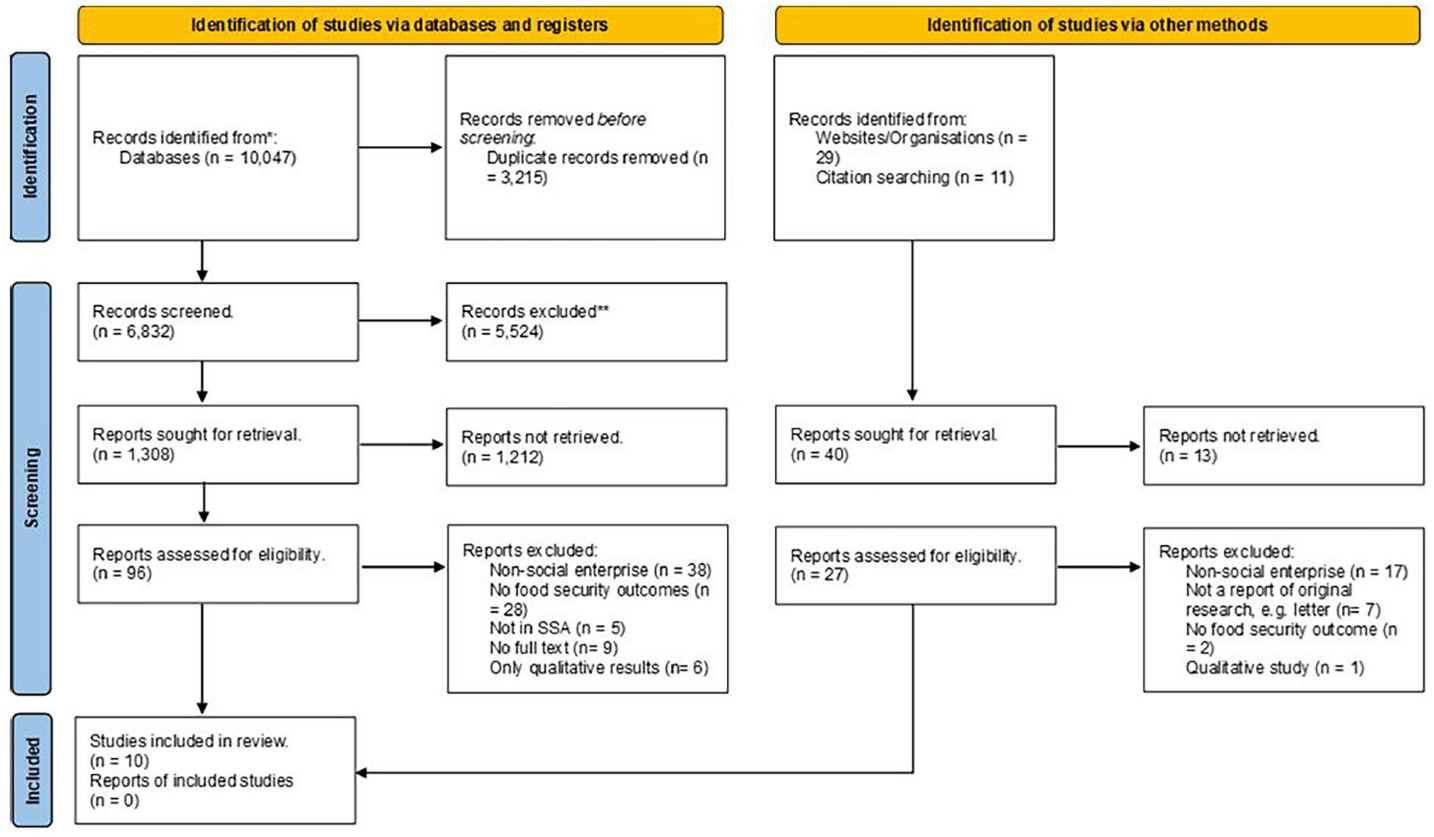
PRISMA flow diagram version 2020 (30).

A total of 10,087 citations were retrieved from academic (*n* = 10,047 studies) and gray literature (*n* = 40) sources. Additionally, 11 studies were retrieved following citation searches, making a total of 10,098 studies. Of the 10,098 citations retrieved, 3,215 were removed as duplicates. Of the remaining 6,883, a further 5,524 citations were removed after screening their titles, as they did not meet the titles eligibility requirements for inclusion. An additional 1,212 citations were removed following abstract screening, leaving 147 articles for full texts review. The full texts screening led to the exclusion of a further 137 citations. Thus 10 papers met the full texts inclusion criteria and were reviewed. The screening process is illustrated in the PRISMA flow diagram (Figure 1).

### Study characteristics

#### Study setting

The studies identified in the search were conducted across eight countries in SSA: Burkina Faso, Ethiopia, Ghana, Kenya, Mozambique, Senegal, Tanzania, and Zambia. Two studies were conducted in Ghana (7, 8), two in Ethiopia (9, 10), and one each in Burkina Faso (11), Mozambique (12), Tanzania (13), Zambia (14), Kenya (15) and Senegal (16). The studies were all published between 2003 and 2019. We have described the study characteristics in Table 3.

**Table 3:** Study characteristics.

First Author	Year	Country	Intervention context	Funding	Implementation by	Type of intervention	Study design	Participants (age, gender, sample size)
Abubakari, A	2014	Ghana	Developmental - Community based	Internally funded	NGO	Micro-finance program	Control - Comparative	N = 180, Mothers of selected VSLA and non-VSLA households, aged 15–44 years, 75% of participants were Muslims
Ayele Z.	2003	Ethiopia	Developmental - Community-based	Internally funded	NGO	Dairy Goat Project: Income generating activities (women groups), local goat credit, saving associations	Before-and-after	Gorogutu District: 100 households belonging to five women’s groups (24% of women in district), in 39 households on availability and consumption of animal source foods by children, 2. Gursum District: 110 households (51% of project beneficiaries), in 25 households examined on milk purchasing practices and milk consumption
Becquey, E.	2022	Burkina Faso	Developmental - community based	Donor funded	NGO together with other local NGOs	Livestock production, poultry revenue generation, women empowerment	Cluster randomized controlled trial	1,054 households with index child and caregiver in 45 communes across 3 regions
Brunie, A	2014	Mozambique	Developmental	Donor funded	NGO	Village Saving and Loan (VSL) groups, rotating labor scheme	Before-and-after	1,276 households
Doocy, S	2005	Ethiopia	Developmental	Not reported	Not reported	Micro-finance program (community banking, solidarity group lending)	Control- comparative	819 households
Kim, Y.	2019	Tanzania	Developmental	Donor/Government funded	NGO	Income generating activities, Construction/rehabilitation of community assets and water sources, Building/rehabilitation of public infrastructure, Community leadership and capacity building through community-based activities	Control-comparative design	512 households surveyed among 2,456 households (332 households from beneficiary villages, 180 from non-beneficiary villages), five persons in each household
Lewis, D.	2011	Zambia	Integrated conservation and development	Donor Funded	Social Enterprise	Biodiversity conservation, food security, and improvement in livelihoods	Retrospective cohort	232 Heavy resilience households (HHs)
Marquis, G. S.	2015	Ghana	Developmental community based	Not reported	Community Support group	Integrated entrepreneurial and nutrition education intervention with microcredit	Quasi-experimental	Caregivers and their 2-5-year-old children
Muthuuri, P.	2021	Kenya	Developmental community based	Not reported	Community Support group (Women entrepreneurs)	Nutrition intervention (complementary feeding)	Cluster-randomized controlled trial	269 mother–child’s dyads and 300 women social entrepreneurs
Wane, A.	2017	Senegal	Developmental community based	Not reported.	Social Enterprise	Innovations to the milk supply chain / modern dairy supply practices	Longitudinal cross sectional	Quantitative study: 445 households (statistical unit) of LDB milk suppliers

#### Study contexts and implementation

The findings indicate that all evaluated SE interventions were implemented in a developmental context rather than an emergency setting. The delivery of these interventions was categorized into three distinct groups based on the stakeholders responsible for their governance and implementation: (i) Interventions led and delivered solely by independently operating SEs, (ii) Interventions led and delivered by SEs in partnership with NGOs or community support groups, and (iii) Interventions led and delivered by SEs in collaboration with the government or governmental agencies.

In the majority of studies (*n* = 7) reviewed, the interventions were executed by NGOs in cooperation with social enterprises, or with other community support groups. Two studies reported that the interventions were led and implemented independently by the social enterprises themselves (14, 16), while one study documented a direct collaboration between social enterprises and government agencies (13). The funding for these interventions originated either from external donors in 4 out of 10 interventions (11–14), or internal funding provided by the implementing organization in 2 out of 10 interventions (7, 9). In four studies, the specific source of funding was not explicitly disclosed (8, 10, 15, 16).

#### Intervention types

The SEs examined in the studies delivered a diverse array of interventions to mitigate food insecurity. These interventions were classified into three distinct groups: (i) *Livestock production and supply*: This category includes interventions such as poultry farming, dairy production/supply and agricultural training (9, 11, 16); (ii) *Microcredits and loan ventures initiatives*: These initiatives encompass village saving and loan programs, as well as cash and voucher initiatives (7, 8, 10, 12), and (iii) *Biodiversity and education programs*: Interventions in this category focus on promoting biodiversity and providing educational programs related to food security (13–15).

#### Intervention designs

The interventions designs reported across the included studies demonstrated considerable heterogeneity. Two studies were cluster -randomized-controlled trials (11, 15), while two others utilized a pre-post intervention design (9, 12). Additionally, two studies adopted a control-comparative design (7, 10), with another two implemented mixed-method approaches that also incorporated control-comparative designs (13, 16). The remaining studies consisted of one retrospective study (14) and one quasi-experimental longitudinal study (8).

#### Study population

The composition of the study populations exhibited significant variability across the included studies. Some studies employed sampling methods that involved selecting specific individuals from designated target households who benefited from the interventions, while others only reported the number of households included without detailing the number of members to each household participating in the studies. For instance, five studies specifically focused on women and children under the age of five (7–9, 11, 15), whereas the remaining studies differentiate between the beneficiaries of the interventions. Table 3 summarizes the characteristics of the included studies.

## Analysis of key findings and contextual factors that influenced implementation and impact

### Livestock production and supply, agricultural training

Three studies have examined the implementation of livestock production, alongside provision of agricultural training, as interventions aimed at improving nutritional outcomes within the study population (9, 11, 16). There was heterogeneity within the livestock and agricultural farming techniques employed, with goats, poultry and cattle, being the primary livestock discussed. Ayele et al. (9) specifically evaluated the Goat Dairy Development Project, which provided training in goat husbandry and product marketing to women groups in various districts in Ethiopia. This initiative resulted in improved availability and productivity of local goat breeds, leading to increased milk production and sales. Consequently, household income rose, as did milk consumption among children, contributing to a greater increase in the availability and consumption of animal-sourced foods (9). However, despite the increase in milk consumption, the overall dietary quality for children remained inadequate, particularly with regard to protein and mineral intake. Similarly, a project in Burkina Faso, focused on education and training in poultry production, which led to an increase in poultry output and greater availability and consumption of eggs among children (16). Despite this, nutrient adequacy among both caregivers and children remained low throughout the study period, with no significant impact on achieving a minimum acceptable diet. Contributing factors may include limited dietary diversity, cultural preferences, or insufficient complementary interventions, resulting in a limited overall effect on improving food insecurity.

An initiative in rural Senegal aimed at enhancing local dairy production involved training farmers in cattle farming and promoting local milk production. This effort led to the creation of various milk-based products, such as yogurt, and the increase in milk production contributed to a rise in the Food Security Index, primarily through improved food availability (16). Although we decided to focus on the quantitative aspect of this mixed-methods study, its qualitative findings lend a substantial input toward their conclusions on improvements in food security (household milk supplies). Table 4 summarizes the key findings reported in the included studies.

**Table 4:** Summary of findings.

First author	Design	Summary description of intervention	Data collection	Outcome measures	Main findings
Abubakari, A	Control- comparative design	Micro-finance program	Assessment of food security	Food access and food consumption/intake	Food access: food shortages are bigger in non-VSLA households 43.3% VSLA vs. 22.2% non-VSLA. Food intake: Improved food intake pattern in VSLA vs. non VSLA
Ayele Z.	Pre-Post intervention assessment	Dairy Goat Development Project: Income generating activities (women groups), local goat credit, saving associations	Evaluation of dietary intake	Food availability and food consumption	Increased child access and consumption of milk as a result of increased goat rearing and milk from goats. 100 and 275% increment in milk availability to 21 and 67 households, respectively, with a similar rise in available protein and fat
Becquey, E.	Cluster randomized controlled trial	Integrated livestock production, poultry revenue generation, women empowerment	Evaluation of dietary intake	Dietary Diversity, food availability and consumption. Minimum acceptable diet in children aged 6–23 months	Nutrient adequacy was low in all study groups and at all time points in both caregivers and index children, the intervention had no impact on minimum acceptable diet (<15% of children (6–23 months) met the minimum acceptable diet)
Brunie, A	Prospective longitudinal study, pre and post assessment	Village Saving and Loan (VSL) groups (microfinance program), rotating labor scheme (increase production and exchange food)	Assessment of food security	Food availability and access, Household Dietary Diversity Score (HDDS), Individual Dietary Diversity Score (IDDS)	VSL participation resulted in an additional 0.47 month of sufficient food among participating households compared with their matched controls. Increase IDD scores: The average increase in the IDDS was 0.81 units (i.e., food groups) higher for children in households participating in VSLs than among children in matched control households
Doocy, S	Control- comparative design	Micro-finance program (community banking, solidarity group lending)	Food Security assessment	Dietary quality and quantity. Food consumption	Household food security among female client households in Sodo was significantly better than in other comparison groups according to a variety of indicators
Kim, Y.	Mixed method study, control-comparative design	1. Income generating activities 2. Construction/rehabilitation of community assets and water sources 3. Building/rehabilitation of public infrastructure 4. Community leadership and capacity building through community-based activities	Food security questionnaire assessment	Food consumption	The project brought about improvement for the beneficiaries, allowing them to eat 0.372 times more often per day on average following the intervention compared to prior to the intervention. Frequency of food consumption increased for intervention participants: 254 families out of 332 answered that the project had substantially helped house- holders to feed their children
Lewis, D.	Retrospective design	Biodiversity conservation, food security, and improvement in livelihoods	Food security assessment	Food security (food availability)	No statistically significant difference in food insecurity after intervention. However, farmers reported small increases in yield (food availability)
Marquis, G. S.	Quasi-experimental longitudinal design	Integrated entrepreneurial and nutrition education intervention with microcredit	Food security assessment; animal source food consumption	ASF diversity score, availability, accessibility and utilization of ASF	The mean animal source food (ASF) frequency score among the intervention group was 20% higher than the control group (<0.001)
Muthuuri, P	Cluster-randomized controlled trial	Nutrition intervention (complementary feeding)	Food security assessment	Food access	Mothers of children with lower age adjusted Mid Upper Arm Circumference z-scores were 62.5% more likely to purchase food than those with higher *z*-scores [odds ratio = 1.625 (CI: 0.53–4.98)] at 95% confidence interval
Wane, A.	Mixed Methods (Quantitative and Qualitative)	Innovations to the milk supply chain / modern dairy supply practices	Quantitative study Used existing household-level, container-level, and milk production data from databases of households of LDB milk suppliers	Qualitative Studies Food Security Index	Quantitative Study 42% of households classified as either “secure” or “highly secure”

#### Contextual factors

Several factors that influenced the implementation and outcomes of the livestock production and agricultural training intervention, included seasonality, milk collection costs, stakeholder trust, and communication within the value chain among farmers (9, 11, 16). Seasonal variations, particularly during dry season, resulted in limited water and pasture availability, which adversely affected milk production (9, 16). The geographic dispersion of farms and the extensive land required for farming were also identified as potential barriers, as the costs associated with milk collection were higher, thereby reducing overall income. Additionally, access to the local markets and opportunities to sell products played a critical role in determining the level of adherence to the intervention of the target population.

### Microcredits/loan ventures initiatives

Four studies examined the implementation of microfinance schemes or loan-base initiatives aimed at improving food security. Abubakari et al. (7) reported that microfinance programs in rural Ghana significantly improved food access and intake in households participating in the Village Saving and Loan Association (VSLA), see Table 4. Additionally, the nutritional status of children under five showed considerable improvement. The success of the VSLA interventions may be attributed to the increased access to funds from the savings associations, which likely enhanced farm production and strengthened purchasing power. A similar approach was applied in Mozambique, where Village Saving and Loan (VSL) groups were established, alongside rotating labor schemes (12). Participation in the VSL groups resulted in increased food availability among participating households, as well as improved Dietary Diversity Scored compared to control groups (12).

In Ethiopia, a community banking system based on solidarity group lending was implemented in two sites (10). Follow up evaluation studies found no significant differences in household food security or nutritional status among participants. However, in one study location that experienced greater drought and food insecurity, female clients and their children showed improved household diet quality and significantly better nutritional status than comparison groups. In Ghana, a combined microcredit loan initiative, which included weekly nutrition and entrepreneurship education sessions for women with children under the age of five, was evaluated. This intervention led to increased availability, accessibility, and consumption of animal source foods (ASF), which in turn also improved the nutritional status of children aged 2–5 years (8).

#### Contextual factors

Factors that facilitated the implementation and effectiveness of microfinancing and lending programs included education and health conditions identified as potential enablers or limiters. In Ghana, children in households participating in a microfinance program demonstrated improved nutritional status, better general health outcomes, and increased access to education (8). These improvements may, in turn, positively influence food security (7). Additionally, the impact of microfinance programs varied by the client’s gender, with female-led households benefiting more from the intervention than male-led households. Credit provided to women proved to be effective in improving outcomes (10). Barriers to the success of these programs included participants’ literacy levels and cultural beliefs, as well as disparities in water and sanitation facilities between intervention and control groups. However, no significant differences were reported in these areas (10).

### Biodiversity and education programs

Biodiversity projects in Tanzania, as studied by Kim et al. and Muthuuri et al., demonstrated positive impacts on food security by increasing food consumption and dietary diversity through community-based interventions (13, 15). These initiatives were combined with income-generating activities such as small-scale farming, agroforestry, and livestock rearing. Kim et al. (13) reported that the intervention led to an increased frequency of food consumption, with households consuming more nutritious foods, including fruits, vegetables, and protein sources. Similarly, Muthuuri et al. (15) observed that biodiversity projects not only enhanced food availability but also improved dietary diversity among participants. The study also revealed that mothers of children with lower Mid Upper Arm Circumference (MUAC) z-scores (poor nutritional status) were 62.5% more likely to prioritize food purchases compared to those with higher *z*-scores (15). This finding suggests that households with nutritionally vulnerable children were more inclined to allocate their income toward food, likely in response to the immediate need to improve their children’s dietary intake.

Similarly, a conservation project detailed by Lewis et al. focused-on training individuals in sustainable agricultural practices, termed “conservation farming,” and offering alternative livelihood skills, such as beekeeping, to former poachers (14). Crops were selected for their suitability for organic cultivation, their impact on food security, resilience to climate variations, and marketability. Participants were encouraged to diversify income sources. Although no direct reduction in food insecurity was observed, farmers—especially women—reported increased agricultural yields. Despite these successes, the study did not establish a direct link between biodiversity improvements and enhanced food security.

#### Contextual factors

Several contextual factors influenced the implementation and effectiveness of the interventions aimed at improving food security and biodiversity outcomes. Gender dynamics played a significant role, as female participants often reported better yields compared to their male counterparts (14). The selection of crops based on local suitability and marketability contributed to higher productivity, while community engagement allowed participants to choose their crops and diversify income sources, fostering ownership of the project. Access to resources, market access for value-added products, and local cultural practices further impacted the success of these initiatives, emphasizing the need to consider local dynamics and preferences.

## Discussion

### Summary of key findings

The review was conducted to scope the literature on SE interventions delivered in Sub-Saharan African context to address food insecurity. The findings of the review revealed several effective approaches. Most interventions were led by social enterprises in collaboration with local or national NGOs and community support groups. The predominant strategies employed by the SEs were livestock-based interventions, microfinance and loan programs, and education activities. Overall, the interventions positively impacted food security, in particular by increasing food availability, accessibility and consumption. However, improving food security was often not the primary motivation for participation in these programs. Although food consumption frequency increased across the studies, many lacked a detailed assessment of the scale of improvement. While access to food improved, other factors, such as economic constraints, market access, environmental challenges, and seasonality, continued to affect outcomes. The significant heterogeneity in intervention outcomes, combined with the qualitative nature of much of the data, limited the generalizability of the findings. Furthermore, the gray literature reviewed highlighted a substantial number of SEs operating in SSA to tackle food insecurity, but robust data on the monitoring and evaluation of these interventions were often lacking.

### Recipients of interventions

In many interventions, women were the primary recipients, especially in microcredit initiatives and livestock programs. Previous studies have documented gender differences in food security programs in SSA (17). Women in low- and middle-income settings often face greater disadvantages and are typically the primary caretakers of children. Microcredits or savings groups can enhance woman’s financial independence and decision-making power within households (18). For instance, evidence from a systematic review highlights how informal savings groups not only improve food security by facilitating access to resources for purchasing food or starting businesses but also promote social cohesion and empower women to take greater control over household decisions. These groups provide emotional and material support, enhancing resilience and economic opportunities (19).

Similarly, evidence from Lewis et al. (14) indicates that women farmers tend to engage in higher agricultural activity with better yields. Studies by Quisumbing and Maluccio (20) and Doss et al., and others, also support the finding that women farmers often show improved agricultural productivity and food security outcomes (20, 21). However, in many rural areas in SSA, men are regarded as the head of the household and retain decision-making power over food purchases and key household decision (22). This raises the question of whether exclusively targeting women in SE interventions is the most effective approach.

### Barriers and facilitators for implementation

The review identified several barriers and facilitators that influenced the implementation of social enterprise interventions in SSA. Geographic and seasonal factors significantly impact agricultural activities, with seasonality disrupting the availability of resources like food and water (23). Studies by Tumusiime and Matotay highlight how seasonal changes can influence agricultural sustainability and food availability in Tanzania, with similar challenges noted across SSA (23, 24). Furthermore, geographic proximity to markets and infrastructure directly affects intervention outcomes, as do high transportation and distribution costs, which often hinder accessibility and profitability for farmers (25). Access to local markets and securing contracts with supermarkets are critical for intervention success, highlighting the role of market integration in fostering food security (24).

Building and maintaining trust among stakeholders, including local and international partners, was also essential for the effectiveness of SE interventions. Compliance issues and distrust can arise in collaborative efforts, affecting outcomes (23, 26). Effective communication among stakeholders remains crucial to successful implementation, particularly when differing agendas are involved. Local community groups and NGOs often serve as mediators, enhancing collaboration and mitigating compliance issues. Busch and Barkema (27) emphasize the importance of orchestrating multi-stakeholder networks, which help align diverse parties and resources toward shared goals, even in low resource settings.

Finally, the adaptability of SE models is a key determinant of their success and scalability. Research by Lyon et al. (28) shows that fledgling SEs require tailored support systems, including capacity-building initiatives and practical, context-specific guidance, to enhance their resilience and effectiveness.

### Sustainability of social enterprise interventions in SSA

The sustainability of SE interventions hinges on several factors, including financial viability and scalability. While many interventions rely on donor funding or internal resources, their longevity depends on creating mechanisms for self-sustainability and replicating success across regions (29). For an intervention to be considered sustainable, it should have a lasting impact on food security and be scalable to other regions or contexts. Evidence from Busch and Barkema (27) underscores the importance of resource alignment and community engagement in ensuring financial stability and long-term impact. Local ownership of interventions fosters a sense of responsibility and ensures that solutions are tailored to the specific needs of the community, emphasizing the importance of local capacity and community engagement in sustaining agricultural interventions (23). While many of the interventions reviewed applied a community-centered approach, it was unclear to what extend the local communities were involved in the planning and implementation of the interventions. Most of the interventions in this review also did not have specific models in place for scale up. Sustainability also requires that interventions be adaptable to changing conditions and closely tied to improving overall well-being, integrating health outcomes into their goals.

### Monitoring and evaluation challenges

One of the major challenges identified across the studies was the lack of robust monitoring and evaluation processes of the implemented interventions. While reports were available on websites of the respective enterprises, comprehensive data and analysis were often missing. Significant gaps existed in the reporting and evaluation of both the processes and outcomes of SE interventions. This lack of comprehensive reporting limits the ability to fully assess the effectiveness of interventions and understand their impact across diverse settings.

### Strength and limitations

This is the first review to our knowledge looking at the impact of SE interventions on food insecurity in SSA. A key strength of this review is the rigorous and systematic search process, which included both peer-reviewed databases and gray literature, ensuring that a wide array of relevant literature has been captured. However, the limited number of studies included and the significant heterogeneity between them posed challenges in making direct comparisons of outcomes. This variability, both in the types of interventions and their reporting, restricted the ability to draw conclusive generalizations about the effectiveness of SE interventions. Furthermore, the small sample of studies may not adequately capture the full diversity of SE interventions across Sub-Saharan Africa. Finally, our searches were conducted in English, and we included only studies published in English. As a result, studies published in other languages, such as French and Portuguese, may have been overlooked.

### Implication of the review findings

While many social enterprise (SE) interventions show promise in addressing food insecurity, there is a notable lack of robust and consistent data on both the implementation processes and evaluation outcomes of these initiatives, as reflected in our findings. To address this gap, it is essential to encourage SEs to adopt rigorous evaluation frameworks and publish detailed findings, ideally in peer-reviewed journals. This can be facilitated by involving researchers and academics from the early stages of conceptualisation through to implementation and evaluation.

Given the heightened vulnerability of the older adults population to food insecurity, and their limited representation in the reviewed studies, it is clear that this group has been largely overlooked in existing research. Future studies should prioritize examining the impact of SE interventions on this underserved population to ensure inclusivity in program design and delivery. Moreover, reporting of SE intervention outcomes should be more uniform to enable meaningful cross-study comparisons. The development and adoption of standardized methods for evaluating and reporting on SE interventions are strongly recommended. Standardization would enhance comparability across studies, which is currently constrained by the wide variation in reporting practices and outcome measures.

Given SEs’ demonstrated capacity to engage communities, tailor interventions to local contexts, and address multiple dimensions of food insecurity, including availability, accessibility, and consumption, there is a strong case for their formal integration into national food and nutrition security strategies. Governments should develop policy frameworks that recognize and support the role of SEs through increased access to funding, capacity-building initiatives, and inclusion in multi-stakeholder platforms for program design and implementation.

While many SE interventions have targeted women, particularly through microfinance and livestock-based programs, yielding positive outcomes in food security, empowerment, and social cohesion, an exclusive focus on women may overlook important household dynamics. To improve effectiveness, interventions should adopt gender-transformative approaches that engage both men and women, address intra-household power relations, and consider the roles and decision-making patterns of all household members. Integrating gender analysis into the planning and evaluation stages of SE programs will help ensure more equitable and sustainable outcomes.

Finally, given the limited number of peer-reviewed publications currently available on this topic, we do not recommend conducting a systematic review or meta-analysis at this stage. Instead, priority should be given to generating high-quality evaluation studies to build a more robust and credible evidence base, which will eventually support future comprehensive reviews and policy development.

## Conclusion

Social enterprises present a promising avenue for mitigating food insecurity in SSA. These initiatives often operate in collaboration with community support groups, NGOs and governmental organizations, enhancing their potential for success and bringing uniting communities around shared goals. The long-term sustainability of these interventions depends on several factors, including financial viability, local ownership, adaptability to changing conditions, integration with local systems, and effective monitoring and evaluation. Addressing these factors will be critical in ensuring that SE interventions contribute to sustainable and lasting improvements on food security in Sub-Saharan Africa.
